# The role of the erythrocyte in the outcome of pregnancy with preeclampsia

**DOI:** 10.1371/journal.pone.0212763

**Published:** 2019-03-06

**Authors:** Márcia Aires Rodrigues de Freitas, Alice Vieira da Costa, Luciana Alves Medeiros, Lucas Moreira Cunha, Ubirajara Coutinho Filho, Mario da Silva Garrote Filho, Angélica Lemos Debs Diniz, Nilson Penha-Silva

**Affiliations:** 1 Department of Gynecology and Obstetrics, Faculty of Medicine, Federal University of Uberlândia, Uberlândia, MG, Brazil; 2 Institute of Biotechnology, Federal University of Uberlândia, Uberlândia, MG, Brazil; 3 Faculty of Chemical Engineering, Federal University of Uberlândia, Uberlândia, MG, Brazil; Nagoya University, JAPAN

## Abstract

The objective of this study was to analyze the relationships of osmotic and mechanical stability of erythrocytes with anthropometric, biochemical, hematologic and hemodynamic variables in pregnant women with preeclampsia (PE). The studied population consisted of 20 normotensive patients and 16 patients with PE. Patients with PE presented worse gestational outcome, greater hematologic impairment, erythrocytes osmotically more stable *in vitro*, but in conditions of isotonicity with the *in vivo* medium, in addition to hyperflow in orbital territory, when compared to normotensive patients. The correlation analysis between anthropometric, hematologic and hemodynamic variables in patients with PE indicated that erythrocytes with lower volumes and lower levels of hemoglobin favor the occurrence of a better gestational outcome, because they are more stable and because they are associated with a decrease in the hemodynamic changes present in the disease. This should mean that the tendency to microcytosis, probably due to a mechanism of compensatory mechanical selection, is a desirable characteristic in the disease.

## Introduction

Pre-eclampsia (PE) is a complex systemic syndrome that occurs after the 20th week of gestation [1] and is characterized by the occurrence of hypertension and/or proteinuria and/or organ dysfunction in a previously normotensive woman [2]. PE is one of the leading causes of maternal and fetal morbidity and mortality in developing countries [3]. Cerebral injury, due to cerebral hyperflow [4–6], dysregulation of the blood-brain barrier and tissue edema are factors directly associated with maternal death [6].

The hemodynamic profile of the Central Nervous System in pregnant women can be observed by Dopplervelocimetric analysis of the ophthalmic artery [7]. The orbital vessels present embryological, anatomical and behavioral characteristics similar to the vessels of the cerebral microcirculation [8]. Therefore, the findings obtained by the ophthalmic artery dopplervelocimetric analysis can be extrapolated to the cerebral circulation [7]. Since 1995, several authors have described the pattern of hyperflow in orbital territory in patients with pre-eclampsia by Doppler velocimetry of the ophthalmic artery [8–11].

Preeclampsia (PE) is described as a hyperdynamic disease associated with diffuse endothelial lesion [12–15]. The preclinical phase of the disease is characterized by a state of high cardiac output and low peripheral resistance (high output, low resistance state) and in the clinical phase of the disease, the opposite occurs (low output, high resistance state) [14,16].

The erythrocytes, the most abundant cells in the bloodstream, suffer greatly from the effects of PE. The disease is associated with important changes in the erythrocyte morphology [17], with early degeneration and de-structuring of its membrane, leading to lysis in the bloodstream [18,19].

For the good performance of its functions, the erythrocyte must remain intact. The red blood cell's ability to maintain the physical and chemical integrity of its membrane in adverse situations is called stability [20,21]. In this sense, the ability of the biological membrane to resist fragmentation in the face of mechanical aggression promoted by the blood flow itself and the friction with the wall of the blood vessels constitutes the so-called mechanical stability, while its ability to remain intact due to volume expansion in a hyposmotic environment is called osmotic stability [22].

Although the evaluation of osmotic stability of the erythrocyte membrane is well established in the literature [23,24], the number of studies on osmotic stability of erythrocyte membrane in preeclampsia is still quite small [11,21,25].

The mechanical stability of erythrocytes also deserves attention [26–28], especially for the analysis of erythrocyte membrane behavior in situations of increased mechanical aggression, which is the main challenge found by red cells in the blood circulation of women with preeclampsia.

The objective of this study was to analyze the osmotic and mechanical stability of erythrocytes and to relate stability variables to anthropometric, biochemical, hematologic and hemodynamic variables in women with preeclampsia, in order to better understand the mechanisms associated with this syndrome.

## Materials and methods

### Population and ethics

The cross-sectional study presented here was previously approved by the Research Ethics Committee of the Federal University of Uberlândia and registered under number CAAE 23236614.4.000.5152. All study participants signed a Free and Informed Consent Form.

This study included 36 women in the third trimester of pregnancy admitted to the Clinical Hospital of the Federal University of Uberlândia between December 2014 and June 2017. Patients without clinical and obstetric intercurrences, and without signs of labor, were included in the control or normotensive group (n = 20). Sixteen patients diagnosed with preeclampsia were included in the study group.

The diagnosis of pre-eclampsia was defined according to the American College of Obstetricians and Gynecologists’ Task Force on Hypertension in Pregnancy [29].

All pregnant women included in the study made supplementation of 5 mg/day of folic acid, during the first trimester of gestation, and 200 mg/day of ferrous sulfate, a dose equivalent to 40 mg of elemental iron per day, for primary prevention of anemia caused by iron deficiency.

Pregnant women with thyroid diseases, erythrocytopathies, chronic hypertension, autoimmune diseases, diabetes, kidney diseases and current use of anticonvulsants, antidepressants, tobacco, alcohol and/or drugs of abuse, and patients with gestations associated with congenital infections (toxoplasma and cytomegalovirus), twinning, fetal malformation and natimortality were excluded from the study. Patients who were on magnesium sulfate and antihypertensive drugs, except methyldopa, at the time of hospital admission were excluded from the study.

### Blood collection

Blood samples (4 mL) were collected by intravenous puncture in an antecubital fossa vein, preferably from the median antecubital vein or the median cephalic vein, directly in two tubes (Vacutainer; Becton Dickinson, Juiz de Fora, MG, Brazil) containing K_3_EDTA (for hematologic analysis and determination of osmotic and mechanical fragility of erythrocytes) and in one tube without anticoagulant (for biochemical analyzes).

### Determination of the osmotic stability of erythrocytes

A duplicate series of test mini-tubes containing 1 mL of 0.1–1.5 g/dL NaCl solution (Labsynth, Diadema, SP, Brazil) was preincubated for 10 minutes in a thermostated bath at 37°C (Marconi, model MA 184, Piracicaba, SP, Brazil). After adding 20 μL of whole blood, the tubes were homogenized and again incubated at 37°C for 30 minutes. The tubes were then centrifuged at 1500 x *g* (Hitachi Koki, model CFR15XRII, Hitachinaka, Japan) for 10 minutes. Supernatants from all tubes were removed and analyzed at 540 nm (A_540_) on a UV-VIS spectrophotometer (Shimadzu, model UV1650TC, Japan) to estimate the amount of hemoglobin released in the erythrocytes lysis.

The graphs of A_540_ as a function of the NaCl (X) concentration were adjusted by sigmoidal nonlinear regression, according to the Boltzmann equation:

$$A_{540}=\frac{A_{\max}-A_{\min}}{1+e^{\left(X-H_{50}\right)/dX}}+A_{\min}$$ (1)

where A_max_ and A_min_ represent respectively the maximum and minimum plateaus of A_540_, H_50_ is the concentration of NaCl capable of promoting 50% hemolysis and dX represents one-fourth of the variation in NaCl concentration responsible for promoting 100% hemolysis [20,24]. The saline concentration at the initial point of the curve defines the variable H_0_, which is the saline concentration required to initiate in vitro hemolysis, and can be calculated by the formula H_0_ = H_50_ + 4dX/2. The saline concentration at the point where the in vitro lysis reaches its maximum plateau defines the variable H_100_, which represents the saline concentration necessary to promote total lysis of the red blood cells, being calculated by the formula H_100_ = H_50_-4dX/2 [11].

### Kinetics of mechanical lysis of erythrocytes

Volumes of 15 mL of the suspension consisting of the mixture of 250 μL of whole blood and 49.75 mL of 0.9 g/dL NaCl solution were inserted into the equipment analysis cell and subjected to mechanical aggression promoted by a propeller under 8000 rpm for 6 minutes at room temperature. Every 30 seconds of stirring, duplicated aliquots of 1 mL suspension were removed and added to minitubes. After centrifugation at 1600 x *g* for 10 minutes in a Hitachi Koki centrifuge (model CFR15XRII, Hitachinaka, Japan), the supernatants were subjected to absorbance readings at 540 nm (A_540_) on a UV-VIS spectrophotometer (Shimadzu, model UV1650TC, Japan) for estimation of the amount of hemoglobin released in the mechanical lysis of erythrocytes.

The graph of the absorbance at A_540_ (A) versus time (t) was adjusted to the hyperbola given by Michaelis-Menten equation [30]:

$$A=\frac{A_{\mathrm{mmax}}t}{t_{1/2}+t}$$ (2)

where A_mmax_ is the maximum plateau of A_540_, which represents the maximum amount of hemoglobin released in the lysis of the entire erythrocyte population, and t_1/2_ is the time interval necessary for promotion of 50% lysis of erythrocytes (A_mmax_/2).

### Doppler ultrasonography of the ophthalmic artery

The Doppler ultrasound of the ophthalmic artery (OA) was performed in Medison (SonoAce 8800MT model, Japan) equipment, by a single examiner with over 10 years of experience. The AO was insonated in the medial region of the optic nerve with linear transducer at 7–10 MHz frequency, 50 Hz filter, 5 kHz pulse repetition frequency and 7 mm sample volume [31]. Only one eye was examined because previous studies did not show significant differences between the eyes [7,8].

### Determination of hematologic and biochemical parameters

An automated system (Sysmex K4500; Sysmex Corporation, Mundelein, IL, USA) was used to obtain the hematologic parameters: red blood cell (RBC) and platelet (Plt) counts, mean platelet volume (MPV), reticulocytes index (Rtc), hematocrit (Ht), hemoglobin (Hb), mean corpuscular volume (MCV), mean corpuscular hemoglobin (MCH), mean corpuscular hemoglobin concentration (MCHC) and red cell distribution width (RDW).

An automated analyzer (Architect c8000, IL, USA) was used to determine the following biochemical parameters: total cholesterol (t-C), triglycerides (TGC), high-density lipoprotein cholesterol (HDL-C), low-density lipoprotein cholesterol (LDL-C), very-low-density lipoprotein cholesterol (VLDL-C), lactate dehydrogenase (LDH), aspartate aminotransferase (AST), alanine aminotransferase (ALT), urea (U), creatinine (Cn), uric acid (UA), human serum albumin (HSA), sodium (Na^+^), potassium (K^+^), indirect bilirubin (i-B), total bilirubin (t-B), serum iron (Fe) and ferritin (Ferritin).

Urine reagent tapes (Labtest, Lagoa Santa, MG, Brazil) were used to evaluate proteinuria.

Reference values described for the third trimester of gestation [32] were used for: RBC, 2.71–4.43 x 10^6^ cells/mm^3^; Hb, 9.5–15.0 g/dL; Ht, 28.0-40.0%; MCV, 81–99 fL; MCH, 29–32 pg/cell; MCHC, 31–36 g%; RDW, 12.7–15.3%; Rtc, 0,5–2%; Plt, 146–429 x 10^3^ cells/mm^3^; MPV, 8.2–10.4 fL; t-C, 219–349 mg/dL; HDL-C, 48–87 mg/dL; LDL-C, 101–224 mg/dL; VLDL-C, 21–36 mg/dL; TGC, 131–453 mg/dL; U, 3–11 mg/dL; Cn, 0.4–0.9 mg/dL; UA, 3.1–6.3 mg/dL; AST, 4–32 U/L; ALT, 2–25 U/L; Na^+^, 130–148 mEq/L; K^+^, 3.3–5.1 mEq/L; LDH, 82–524 U/L; i-B, 0.1–0.5 mg/dL; t-B, < 0.1–1.1 mg/dL; Fe, 30-193 μg/dL; Ferritin, 0–116 ng/mL; HAS, 2.3–4.2 g/dL.

#### Statistical analysis

Categorical variables were compared with the use of the chi-square test. The normality of the data was evaluated by the Shapiro-Wilk test. The Student's t-test and the Mann-Whitney test were used to compare between groups the results with normal and non-normal distribution, respectively. Most of the variables did not present normal distribution and therefore the Spearman correlation analysis was used to determine the associations between the anthropometric, hemodynamic, hematologic, and biochemical variables in the group of volunteers with preeclampsia. Correlations with values of p <0.05 were considered significant. All analyzes were performed using Origin 8.5 (Microcal, Northampton, MA, EUA) and SPSS 15.0 (SPSS Inc., Chicago, IL, USA).

## Results

**Fig 1:**
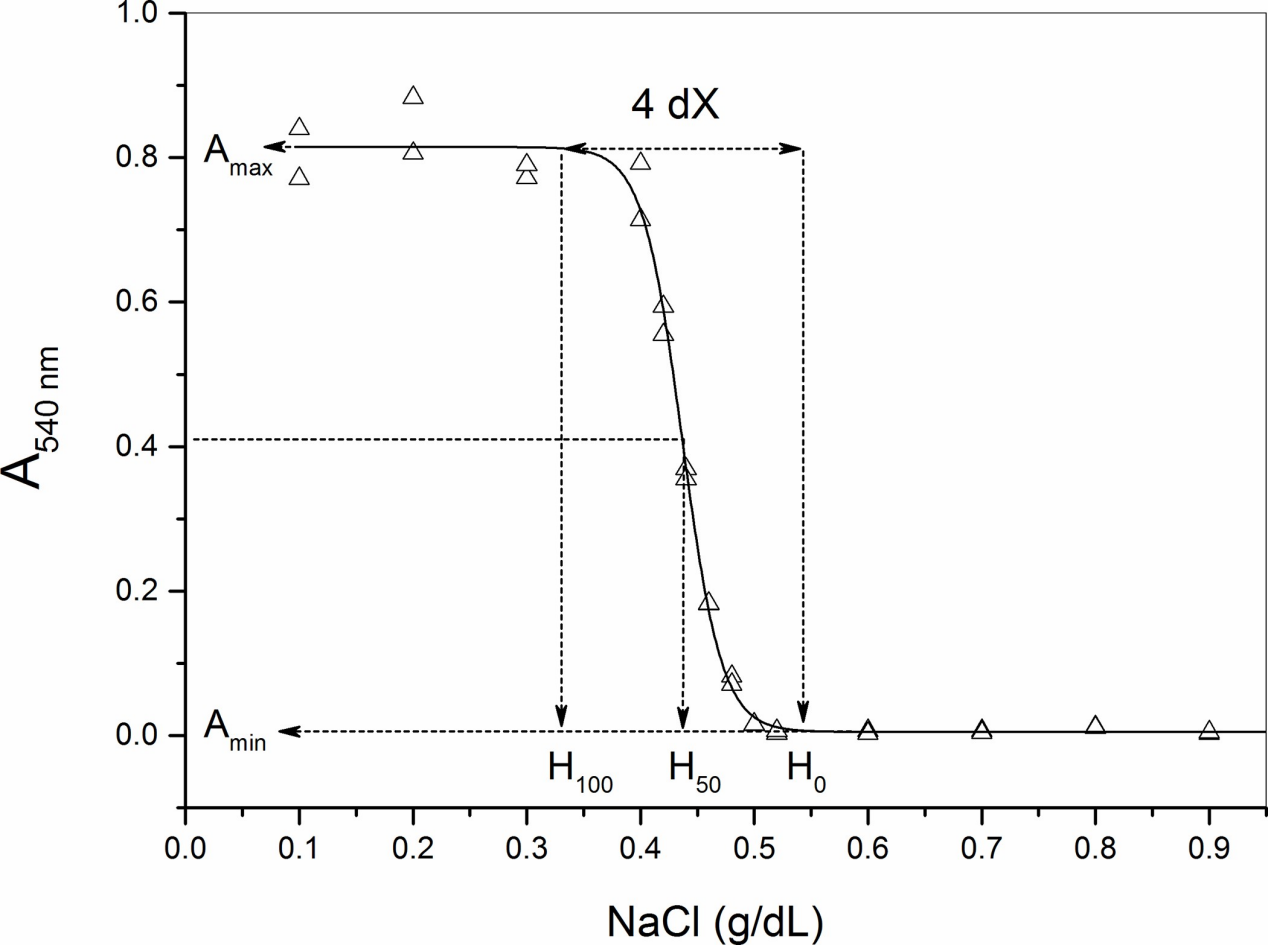
Typical curve of hyposmotic lysis of erythrocytes, with definition of the osmotic stability variables A_mim_, A_max_, H_0_, H_50_, H_100,_ and dX.

**Fig 1** shows a typical sigmoid curve obtained in the determination of erythrocyte membrane stability variables. A blood isotonicity condition occurs in the region to the right of the curve, where the value on the ordinate of the lower plateau of the curve defines the variable A_min_, which represents the amount of basal hemolysis present in the blood sample of each volunteer in the study. The higher this A_min_ value, the lower the erythrocyte stability under osmotic conditions similar to those observed *in vivo*. Therefore, A_min_ is the osmotic stability variable that most represents the stability of erythrocytes *in vivo*. Following this curve, from right to left, as it shall be seen, the salt concentration where the lysis begins to occur defines the variable H_0_. With the decrease in saline concentration, the lysis rate will reach 50% of the maximum lysis at the intermediate point of the curve, whose value in the abscissa defines the variable H_50_. When the lysis rate reaches its maximum point, which occurs when the curve reaches the upper plateau, the salt concentration where the lysis rate runs out defines the variable H_100_. The variables H_0_, H_50_ and H_100_ are proportional to the osmotic fragility of the erythrocytes. Therefore, the use of their inverse forms, 1/H_0_, 1/H_50_ and 1/H_100_ is that it effectively represents the osmotic stability of those cells. The ordinate value of the upper sigmoid plateau defines the variable A_max_, which represents the maximum possible rate of lysis of the erythrocytes of the analyzed blood sample and which therefore must show obvious associations with hemoglobin levels, erythrocyte counts and the hematocrit values of the donor of the analyzed blood sample. There is yet another primary variable defined by the mathematical model of statistical fit itself; this variable is dX, which represents a quarter of the variation in saline concentration required to promote 100% hemolysis. dX is effectively a variable of osmotic stability of erythrocytes and, therefore, the dX/H_50_ and dX/A_min_ ratios are directly proportional to the erythrocyte membrane stability, since A_min_ and H_50_ have inverse relationships with the stability of these cells.

**Fig 2:**
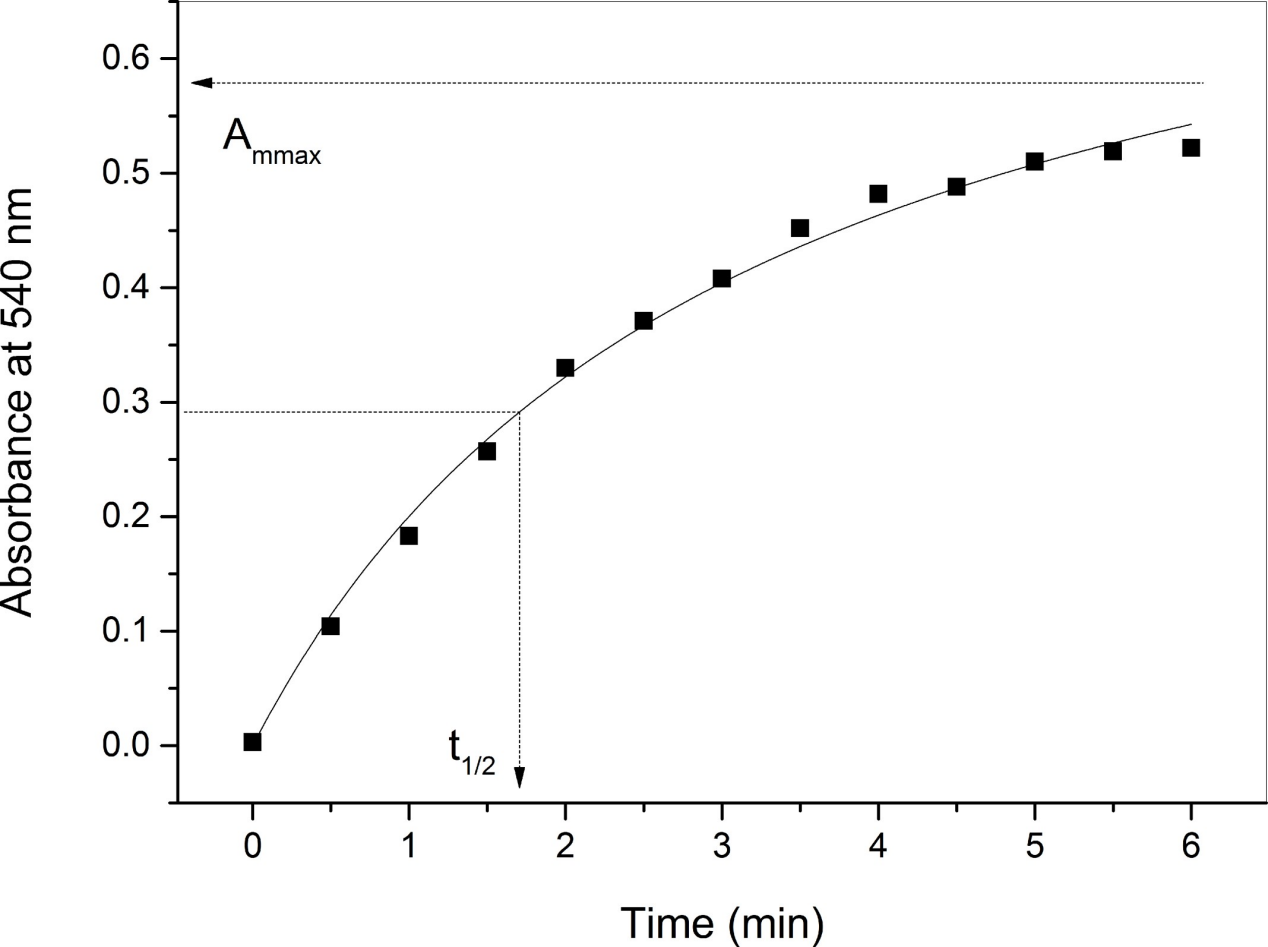
Typical curve obtained in the kinetics of erythrocyte mechanical lysis, with definition of the kinetic variables t_1/2_ and A_mmax_.

**Fig 2** shows a mechanical lysis curve of erythrocytes. With increasing time under mechanical stress, erythrocytes undergo lysis and release hemoglobin, resulting in increased absorbance by 540nm. The data fit defines a hyperbolic curve whose upper plateau defines the A_mmax_ variable, which represents the absorbance associated with the amount of hemoglobin released when there is 100% lysis of the erythrocyte population. The curve passes through an intermediate point, whose abscissa value (t_1/2_) represents the time required to promote 50% hemolysis. The t_1/2_ variable is directly related to the mechanical stability of erythrocytes.

**Fig 3:**
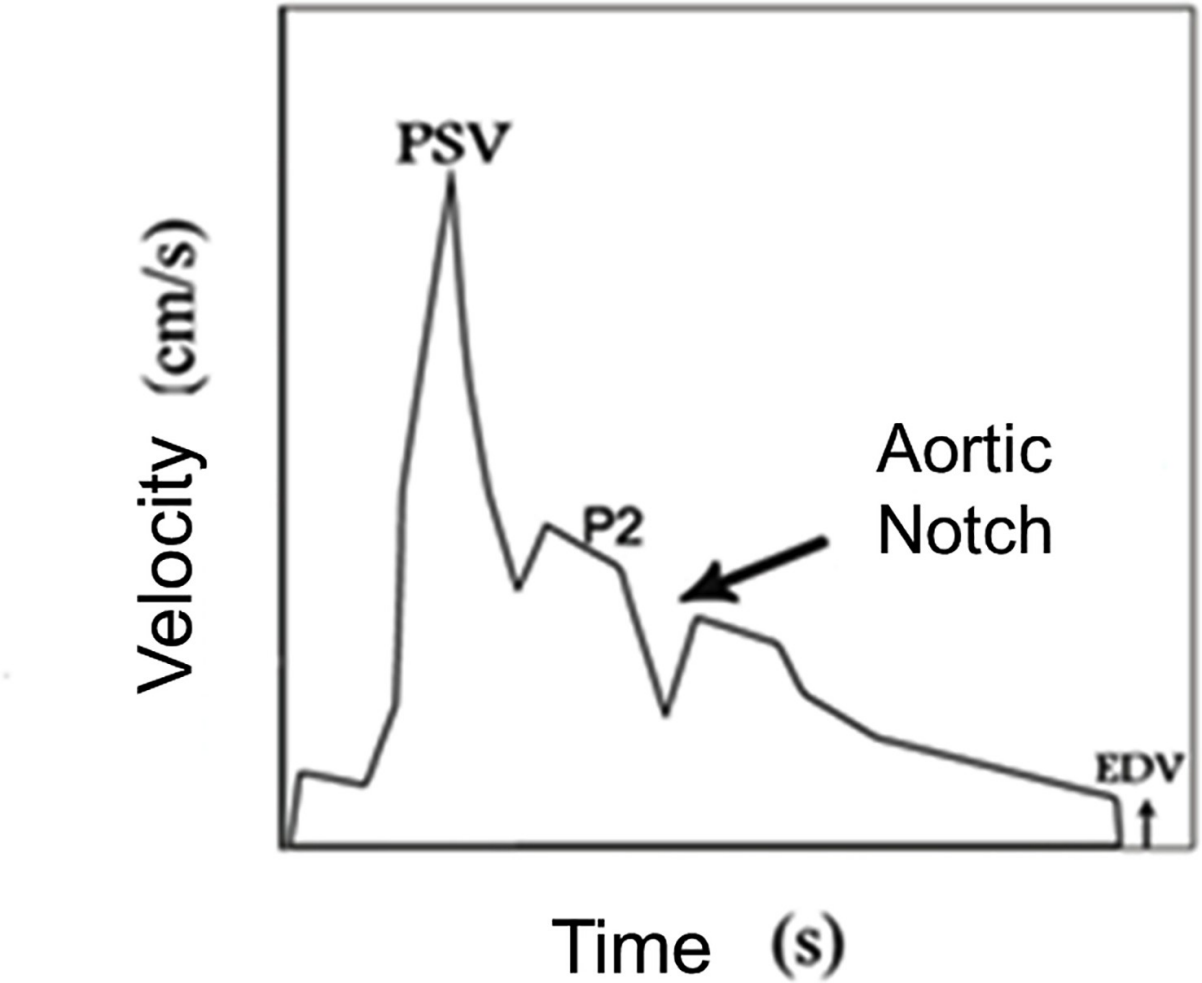
Doppler velocimetry of the ophthalmic artery of a pregnant woman with preeclampsia. The Y axis represents the flow velocity (cm/s) and the X axis represents time (seconds). A rapid increase in velocity, with formation of the peak systolic velocity (PSV), is followed by a rapid fall and a new velocity rise, with the formation of the second systolic peak (P2), followed by the aortic notch, which closes the systolic cycle. The subsequent rise of velocity constitutes the diastolic phase of the pulse wave velocity (PWV), which ends with the end-diastolic velocity (EDV). In severe preeclampsia there is an increase in the amplitude of P2, characterizing the presence of a hump.

**Fig 3** is a schematic representation of the variation in blood flow velocity in the ophthalmic artery (OA) as a function of the duration time of a cardiac cycle. At each ejection of blood from the left ventricle (systole), a pulse pressure occurs which is represented by a rapid upward curve, generating the Systolic Velocity Peak (PSV), which is followed by sudden drop and a secondary elevation of velocity, generating a second systolic peak (P2) and a notch (aortic notch) before the end of the systolic cycle. As the vascular diameter returns to normal, the accumulated energy provides a potential necessary to promote continuous flow during diastole, which is represented in the graph by a new rise in velocity that is followed by slow deceleration, ending in the peak that characterizes the end-diastolic velocity (EDV).

The interpretation of the graphical representation of the OA Doppler occurs by analysis of 1) the shape of the pulse velocity wave (PWV), 2) the velocity peaks and 3) the average velocity of a cardiac cycle (V_mean_), as well as the Pulsatility Index (PI), given by (PSV-EDV)/V_mean_, the Resistance Index (IR), given by (PSV-EDV)/PSV, and the Peak Ratio (PR), given by P2/PSV. In pre-eclampsia, there is a specific alteration in the morphology of the wave, which acquires the shape of hump, due to the increase in amplitude and rounding of P2, indicating the presence of hyperflow in orbital territory. The elevation of PR and P2 also indicates hyperflow in orbital territory. In contrast, the decrease in IP and IR represents an increase in the flow velocity at that site [8,33].

**Table 1** shows baseline characteristics of the population of pregnant women with PE in this study.

**Table 1:** Baseline characteristics of the study population.

Parameters		Normotensive (N = 20)	Preeclampsia (N = 16)	p
Maternal Age (years)	†	25.60 ± 6.36 (20)	26.19 ± 7.92 (16)	0.811
Ethnic Group Distribution	^§^			0.648
White		65% (13)	50.0% (8)	
Mixed		20% (4)	31.3% (5)	
Black		15% (3)	18.8% (3)	
Weight Gain (kg)	*	10 (10–11) (17)	15.5 (14.38–20) (8)	0.002
Parity	^§^			0.119
Nulliparous		21.05% (4)	50% (8)	
Multiparous		78.95% (15)	50% (8)	
Body Mass Index (kg/m^2^)				
Before Pregnancy	†	24.70 ± 4.80 (19)	26.63 ± 4.72 (7)	0.367
At the end of pregnancy	*	29.7 (27.9–31.5) (18)	33.8 (30.6–39.0) (13)	0.042
Gestacional Age (weeks)	*	40 (39.5–40.5) (20)	37.5 (35–39) (15)	0.001
Birth Weight (kg)	†	3.6 ± 0.4 (19)	2.7 ± 0.9 (15)	<0.001
Placenta Weight (g)	*	590 (555–685) (19)	490 (417–597.5) (16)	0.010
Delivery Type	^§^			0.504
Cesarean		78.9% (15)	87.5% (14)	
Vaginal Birth		21.1% (4)	12.5% (2)	
Apgar Score at 1 minute	^§^			0.428
1			7.7% (1)	
4				
5				
6				
7			7.7% (1)	
8		66.7% (12)	53.8% (7)	
9		33.3% (6)	23.1% (3)	
10			7.7% (1)	
Apgar Score at 5 minute	^§^			0.940
8		11.1% (2)	14.3% (2)	
9		72.2% (13)	64.3% (9)	
10		16.7% (3)	21.4% (3)	
Sex of the Newborn	^§^			0.179
Male		65% (13)	40% (6)	
Female		35% (7)	60% (9)	
Blood Group	^§^			0.475
A (–)		5.0% (1)	6.3% (1)	
A (+)		20% (4)	43.8% (7)	
B (–)		-		
B (+)		10% (2)	18.8% (3)	
O (–)		10% (2)	6.3% (1)	
O (+)		50% (10)	25% (4)	
AB (–)		-		
AB (+)		5% (1)		
Systolic Blood Pressure (mmHg)	*	120 (110–120) (19)	155 (140–165) (16)	0.001
Diastolic Blood Pressure (mmHg)	*	75 (70–80) (19)	100 (90–110) (16)	0.001
Red Blood Cell Count (10^6^ cells/mm^3)^	†	4.08 ± 0.38 (20)	4.11 ± 0.40 (15)	0.991
Hematocrit (%)	†	34.99 ± 2.84 (20)	34.37 ± 3.71 (15)	0.596
Hemoglobin (g/dL)	†	12.03 ± 1.07 (20)	11.73 ± 1.51 (15)	0.521
MCV (fL)	†	86.01 ± 4.85 (20)	83.61 ± 6.32 (15)	0.232
MCH (pg)	†	29.55 ± 2.30 (20)	27.30 ± 4.40 (15)	0.202
MCHC (g/dL)	†	34.17 ± 1.12 (20)	33.57 ± 1.32 (15)	0.168
RDW (%)	†	13.67 ±1.48 (20)	13.80 ± 2.10 (15)	0.603
Reticulocytes Index (%)	*	0.95 (0.4–1.7) (18)	1.4 (0.8–3.1) (15)	0.036
Platelet Count (10^3^ cel/mm^3)^	†	215.0 ± 48.8 (20)	215.7 ± 61.61 (15)	0.968
MPV (fL)	*	11.16 (10.5–12.2) (20)	10.40 (10–12) (15)	0.254
Total Cholesterol (mg/dL)	*	215.9 (184–249) (19)	208.02 (184–232) (14)	0.706
HDL-Cholesterol (mg/dL)	†	58.77 ± 17.28 (18)	65.42 ± 16.79 (16)	0.270
VLDL-Cholesterol (mg/dL)	*	40 ± 15 (19)	40 ± 17 (13)	0.910
LDL-Cholesterol (mg/dL)	†	123 ± 48.36 (19)	121.93 ± 50.49 (13)	0.593
Triglycerides (mg/dL)	*	201.1 (138–229.7) (18)	207.3 (170.8–274) (15)	0.580
Proteinuria	^§^			<0.001
Proteinuria 0+		100% (20)	30% (3)	
Proteinuria 1+			30% (3)	
Proteinuria 2+			30% (3)	
Proteinuria 3+			10% (1)	
Alanine Aminotransferase (U/L)	*	15.1 (13.2–17.8) (20)	19.3 (15.9–26.5) (16)	0.010
Aspartate Aminotransferase (U/L)	*	9.0 (7.8–12.0) (19)	12.4 (11.0–19.5) (16)	0.020
Lactate Dehydrogenase (U/L)	*	185.5 (175–206) (18)	241.5 (209.5–317) (16)	<0.001
Urea (mg/dL)	*	14.2 (12.6–15.8) (20)	24.4 (16.7–30.8) (16)	0.001
Creatinine (mg/dL)	†	0.61 ± 0.13 (20)	0.72 ± 0.23 (16)	0.008
Uric Acid (mg/dL)	*	4.45 (3.85–4.80) (20)	5.8 (5.7–7.2) (16)	<0.001
Sodium (mEq/L)	†	138.06 ± 1.77 (16)	137.48 ± 2.42 (12)	0.492
Potassium (mEq/L)	†	3.99 ± 0.27 (18)	4.29 ± 0.34 (12)	0.021
Indirect Bilirubin (mg/dL)	†	0.25 ± 0.12 (19)	0.21 ± 0.13 (11)	0.437
Total Bilirubin (mg/dL)	†	0.41 ± 0.16 (19)	0.39 ± 0.18 (14)	0.573
Iron (mg/dL)	†	93.66 ± 37.37 (18)	94.46 ± 41.04(15)	0.594
Ferritin (ng/mL)	*	29.1 (20.6–25.66) (17)	39.3 (26.5–106.5) (15)	0.105
Human Serum Albumin (g/dL)	†	3.41 ± 0.27 (18)	3.26 ± 0.4 (12)	0.286
RI	*	0.78 (0.75–0.80) (18)	0.69 (0.64–0.74) (13)	0.020
PI	†	1.93 ± 0.54 (18)	1.51 ± 0.37 (13)	0.017
PSV (cm/s)	*	31.4 (28.5–37.3) (19)	30.6 (26.3–40.1) (13)	0.570
P2 (cm/s)	*	16.5 (14.6–24.3) (19)	23.8 (20.1–24.5) (13)	0.045
PR	†	0.56 ± 0.13 (19)	0.75 ± 0.14 (13)	0.001
V_mean_ (cm/s)	*	12.46 (10.6–17.03) (18)	14.93 (12.73–17.82) (13)	0.125
EDV (cm/s)	*	6.81 (5.98–11.74) (19)	9.88 (7.21–10.43) (12)	0.164
A_min_ (ΔOD)	†	1.086 ± 0.991 (18)	0.968 ± 0.205 (16)	0.048
A_max_ (ΔOD)	*	0.015 (0.004–0.020) (18)	0.013 (0.006–0.023) (16)	0.959
H_o_ (g/dL NaCl)	†	0.476 ± 0.022 (18)	0.470 ± 0.029 (16)	0.515
H_50_ (g/dL NaCl)	*	0.442 (0.432–0.460) (18)	0.431 (0.410–0.452) (16)	0.187
H_100_ (g/dL NaCl)	*	0.423 (0.398–0.427) (18)	0.391 (0.381–0.414) (16)	0.154
1/H_50_ (g/dL NaCl)^-1^	*	2.264 (2.174–2.313) (18)	2.317 (2.210–2.441) (16)	0.187
dX (g/dL NaCl)	*	0.016 (0.013–0.019) (18)	0.020 (0.012–0.026) (16)	0.376
dX/H_50_	*	0.036 (0.029–0.047) (18)	0.042 (0.026–0.059) (16)	0.506
dx/A_min_	*	0.016 (0.012–0.018) (18)	0.017 (0.010–0.032) (16)	0.347
A_mmax_ (ΔOD)	*	0.638 (0.569–0.714) (20)	0.594 (0.503–0.668) (16)	0.336
t_1/2_ (s)	*	1.33 (0.77–2.00) (20)	1.36 (0.99–2.50)(16)	0.404

^§^Qualitative variables, expressed as percentage and compared with use of the chi-squared test.

*Non-normally distributed data, expressed as median (Q1-Q3) and compared with use of the Mann-Whitney test.

†Normally distributed data, expressed as mean ± SD and compared with use of Student t test.

Abbreviations: N, number of participants; MCV, mean corpuscular volume; MCH, mean corpuscular hemoglobin; MCHC, mean corpuscular hemoglobin concentration; RDW, red cell distribution width; MPV, mean platelet volume; HDL, high density lipoprotein; VLDL, very low density lipoprotein; LDL, low density lipoprotein; RI, resistance index; PI, pulsatility index; PSV, peak systolic velocity; P2, second peak of systolic velocity; PR, peak ratio; V_mean_, mean velocity; EDV, end diastolic velocity; A_min_, absorbance at 540 nm associated with residual lysis of the erythrocytes population; A_max_, absorbance at 540 nm associated with lysis of the whole population of erythrocytes; H_0_, saline concentration where *in vitro* hemolysis begins; H_50,_ saline concentration capable of promoting 50% hemolysis_,_ H_100_, saline concentration where *in vitro* lysis ends; 1/H_50_, inverse the NaCl concentration capable of promoting 50% hemolysis; dX, variation in the concentration of NaCl responsible for total hemolysis; A_mmax,_ absorbance at 540 nm associated with the mechanical lysis of the whole population of RBC; t_1/2_, time interval required for mechanical lysis of half of the erythrocyte population.

Regarding the anthropometric variables, the pregnant women with PE had significantly higher values of BMI at the end of gestation in comparison to normotensive pregnant women. In addition, in the group of pregnant women with PE, gestational age, fetal weight and placental weight were significantly lower compared to the control group.

Regarding hematologic variables, the PE group had significantly higher reticulocytes indices than the normotensive group.

Regarding the biochemical variables, the PE group had significantly higher values of blood analytes: urea, creatinine, uric acid and potassium; and also of the enzymes ALT, AST and LDH, compared to the normotensive group.

As for the Dopplervelocimetry of the OA, the PE group showed significantly higher values of P2 and PR, and significantly lower values of PI and RI, when compared to the normotensive group.

As for the osmotic stability variables of the erythrocyte membrane, the PE group had a significantly lower A_min_ value when compared to the normotensive group.

**Fig 4:**
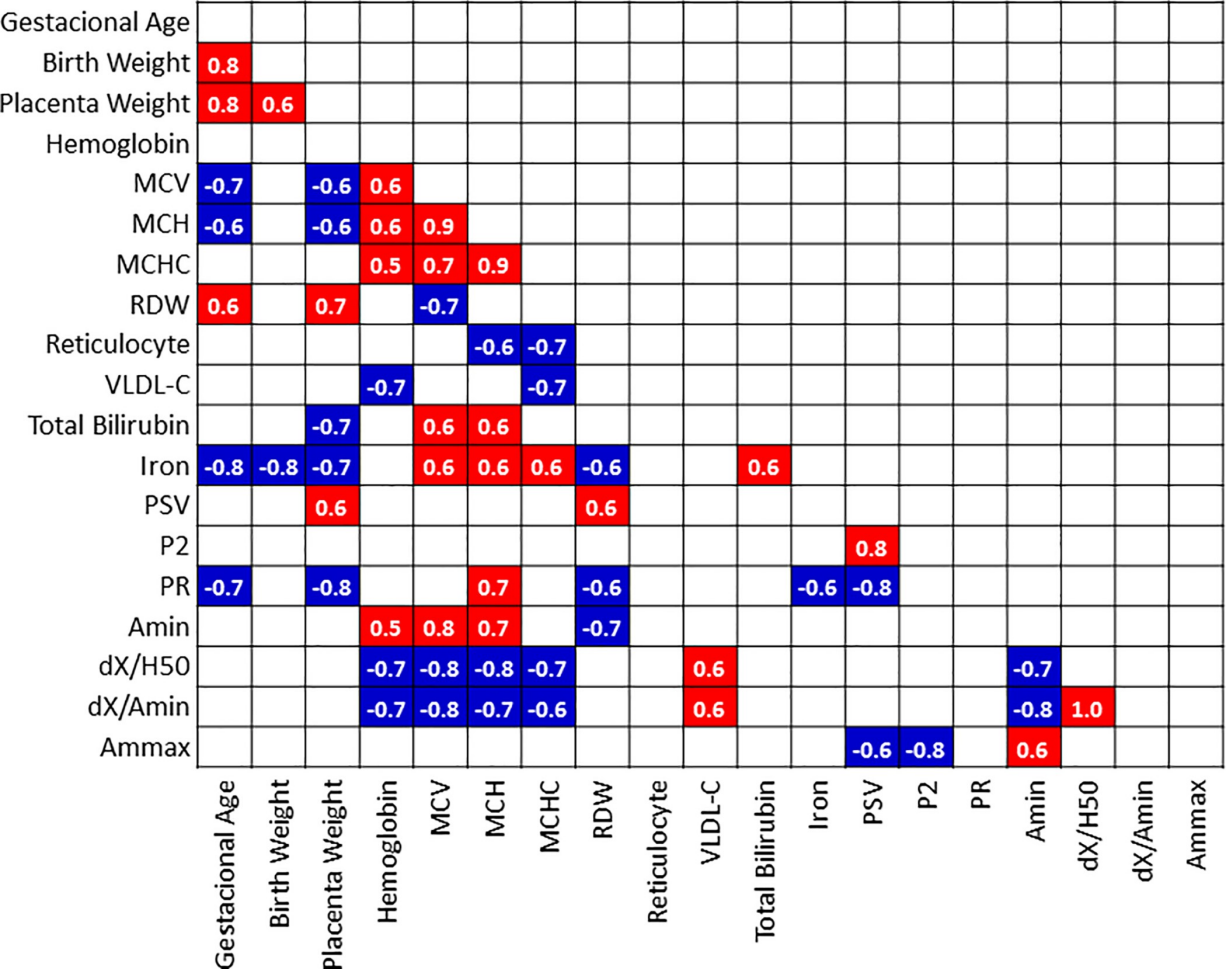
Values of ρ coefficients for significant (p<0.05) Spearman correlations between some pairs of variables in pregnant women with preeclampsia. Red and blue shading were used for positive and negative correlations, respectively. Abbreviations: MCV, mean corpuscular volume; MCH, mean corpuscular hemoglobin; MCHC, mean corpuscular hemoglobin concentration; VLDL-C, very low density lipoprotein cholesterol; PSV, peak systolic velocity; P2, second peak of systolic velocity; PR, peak ratio; A_min_, absorbance at 540 nm associated with residual lysis of the erythrocytes population; dX, variation in the concentration of NaCl responsible for total hemolysis; H_50,_ saline concentration capable of promoting 50% hemolysis; A_mmax,_ absorbance at 540 nm associated with the mechanical lysis of the whole population of RBC.

**Fig 4** presents the Spearman correlation matrix between some pairs of variables considered in this study.

### Some correlations deserve special mention

In relation to the osmotic stability variables, some correlations deserve to be highlighted. The negative correlations observed for dX/A_min_ and dX/H_50_ with MCV and MCH, MCHC and Hb mean that erythrocytes with lower volume and lower hemoglobin content were osmotically more stable. At least for the dX/A_min_ index, this finding was influenced by the variable A_min_, which was effectively lower in the PE group (Table 1). Indeed, A_min_ presented positive correlations with MCV and Hb and MCH; this means that erythrocytes with lower volume and lower hemoglobin levels were more stable even at osmolarity conditions close to those present in *in vivo* conditions. The positive correlations observed for dX/A_min_ and dX/H_50_ with VLDL-C shows that this increased membrane stability was also favored by elevated lipidemia.

Regarding the hematologic variables, some correlations shall be highlighted. The positive correlations of MCV with iron, hemoglobin, MCH and MCHC, on the one hand, and with total bilirubin, on the other hand, should mean that smaller erythrocytes were associated with lower levels of iron and hemoglobin, which is quite obvious, and that smaller erythrocytes were associated with lower rate of clearance and bilirubin production. The negative correlation observed for MCV with placental weight means that larger erythrocytes were associated with worse gestational outcomes. And finally, the positive correlation observed between MCH and the Dopplervelocimetric variable PR sustains the idea that lower levels of hemoglobin make unnecessary the occurrence of cerebral hyperflow.

Regarding the variables of mechanical lysis kinetics, the negative correlation of A_mmax_ with the Doppler velocimetric variables P2 shall be highlighted. It suggests that lower levels of hemoglobin, present in erythrocytes more resistant to both osmotic and mechanical lysis, were associated with lower compensatory cerebral flow.

## Discussion

Preeclampsia (PE) is a heterogeneous disorder that has an important impact on the maternal and fetal organism [34,35]. In this study, the group of pregnant women with preeclampsia presented the worst gestational outcome, with delivery before 39 weeks and lower fetal and placental weight. The presence of higher Body Mass Index (BMI) at the end of gestation in the PE group (Table 1) reinforces the previous findings of association between PE and obesity [36,37]. The significant differences observed between the groups regarding hematologic and biochemical variables are consistent with the multisystemic involvement reported for this disease [38,39].

Normal erythrocytes have a typical biconcave disk shape. This cell geometry provides a larger membrane surface area per cytoplasmic volume, which provides greater deformability in small vessels and maintenance of their physicochemical integrity [40]. Therefore, changes in the erythrocyte format are associated with changes in their properties. Macrocytic erythrocytes, with higher hemoglobin content, tend to acquire more spherical forms, with lower capacity to incorporate water in their interior and greater vulnerability to undergo lysis in hypo-osmotic conditions, i.e., they have lower osmotic stability. On the other hand, microcytic erythrocytes, with lower hemoglobin content, have a greater ability to remain intact under hyposmotic conditions, i.e., they present greater osmotic stability [41].

In this study, the hematologic involvement of the disease was associated with greater osmotic stability of erythrocyte membrane. This has already been demonstrated in other studies [11,18,25,42]. But this study is the first to demonstrate the occurrence of increased erythrocyte membrane stability under conditions of osmolarity that are equivalent to those existing *in vivo*. It is possible that the osmotic stability test may be useful in characterizing the onset and progression of preeclampsia. The involvement of A_min_ in one of these erythrocyte stability indices and the fact that the A_min_ value was lower in the group of pregnant women with PE (Table 1) means that this increased osmotic stability of erythrocytes *in vitro* reflects a greater stability also *in vivo*.

In this study, the ophthalmic arthery (OA) Doppler velocimetry allowed to observe the hyperflow in orbital territory in the group of patients with PE, which supports findings reported in other studies [7,8]. Hyperflow in orbital territory means hyperflow in cerebral territory [6,33,43], which is a pathophysiological mechanism of involvement of central nervous system in this disease and, therefore, constitutes a threat to maternal life [5,33].

This means that effective measures to reduce cerebral hyperflow in the disease are desirable. The negative association observed between A_mmax,_ absorbance at 540 nm associated with the mechanical lysis of the whole population of RBC, and the hemodynamic variable P2, second peak of systolic velocity (Fig 4) suggests that lower levels of hemoglobin are associated with a lower compensatory cerebral flow. Indeed, the positive correlation observed between peak ratio (PR) and mean corpuscular volume (MCH) (Fig 4) also support the idea that lower levels of hemoglobin make unnecessary the occurrence of cerebral hyperflow.

In pregnant women, the hyperdynamic state present in PE is responsible for a higher pulse pressure in the left ventricle [12,14,16]. This situation requires the existence of erythrocytes with greater mechanical resistance to adequately oxygenate the tissues. The significant negative correlations of mean corpuscular volume (MCV) and mean corpuscular hemoglobin (MCH) with gestational age and placental weigh (Fig 4) indicate that, in preeclampsia, erythrocytes with lower volume and lower concentration of hemoglobin favor the occurrence of a better gestational outcome. These findings make sense in the light of the association observed between elevated hematocrit values and fetal complications [44,45], with a higher risk of preterm birth and growth restriction when hematocrit is above 43% between 31 and 34 weeks of gestation [45].

This indicates that the tendency to microcytosis would be a desirable feature in preeclampsia. But why? The answer to this question seems to lie in the negative associations of the stability indices dX/A_min_ and dX/H_50_ with hemoglobin, MCV and MCH. These correlations indicate that erythrocytes with lower volume and lower hemoglobin concentration were more stable in hyposmotic medium *in vitro*, certainly due to their greater capacity to undergo volume expansion prior the occurrence of lysis [18]. The strong positive correlations of MCV with hemoglobin, and mean corpuscular hemoglobin concentration (MCHC) (Fig 4), which are quite obvious, support this inference, since a lower concentration of hemoglobin means less ability to retain water and greater capacity to expand in hyposmotic environment [18].

The involvement of A_min_ in one of these erythrocyte stability indices and the fact that the A_min_ value was lower in the group of pregnant women with PE (Table 1) means that this increased osmotic stability of erythrocytes *in vitro* reflects a greater stability also *in vivo*.

Larger erythrocytes with higher hemoglobin levels offer more resistance to blood flow and are more vulnerable to premature lysis when passing through small diameter capillaries, especially in the face of increased pressure. Indeed, the positive correlations of MCV and MCH with total bilirubin (Fig 4) suggest that erythrocytes with higher volume and higher hemoglobin concentration contributed more effectively to the determination of circulating levels of total bilirubin.

The strong positive correlations of MCV and MCH with A_min_ (Fig 4) reinforce the idea that erythrocytes with greater volumes and higher hemoglobin contents are more likely to undergo hemolysis *in vivo* [11]. Indeed, other studies have shown the predominance of a red cell population with lower MCV values in women who developed pre-eclampsia compared to women without complications in pregnancy [18,46].

It is certainly for this reason that higher levels of iron, which favors the elevation of MCV and MCH, were associated with lower gestational age and placental weight (Fig 4).

The significantly higher reticulocyte count in the PE group (Table 1) is a pathophysiological response to the increase in erythrocyte removal rate. The significant negative correlation of the reticulocyte index with MCH and MCHC (Fig 4) makes a lot of sense with the occurrence of premature removal of erythrocytes with higher hemoglobin content.

As the occurrence of dyslipidemia, which is a condition considered normal in pregnancy [47], is exacerbated in preeclampsia [48,49], it is possible that the erythrocyte behavior is also associated with lipidemia in this pathological condition. The positive correlations presented by the dX/A_min_ and dX/H_50_ with VLDL-Cholesterol (Fig 4) show that elevated lipidemia is allowing the occurrence of increased red blood cell stability. Indeed, an increase in the cholesterol/phospholipid ratio in the erythrocyte membrane contributes to increase its resistance to lysis in hypotonic medium [50–52], which is true especially in view of a higher content of reticulocytes.

In normal pregnancy, under the current practice of nutritional supplementation with ferrous sulfate in the last trimesters of pregnancy, there is a progressive increase in MCV until delivery. This tendency towards macrocytosis produces reduction in the diameter and increase in the sphericity of the erythrocytes. This predisposes to the *in vitro* decrease in erythrocyte membrane resistance to lysis in hyposmotic conditions [18]. The results of the present study show an inverse phenomenon in preeclampsia, indicating the occurrence of disturbance in the process of hemorheologic adaptation to pregnancy. It is possible that in pre-eclampsia the promotion of this tendency to macrocytosis, favored by the supplementation of ferrous sulfate, may not be a desirable goal in view of the hypertensive conditions of the disease, which seem to cause a mechanical selection of erythrocytes with lower volumes and lower concentrations of hemoglobin. However, this is a question that demands further investigation.

## Conclusion

The tendency to microcytosis in preeclampsia seems be due to a compensatory mechanism of mechanical selection that minimizes cerebral hyperflow and favors better outcome in pregnancy.

## Supporting information

S1 File Database. Study database.
(XLSX)
